# Motor imagery training to improve language processing: What are the arguments?

**DOI:** 10.3389/fnhum.2023.982849

**Published:** 2023-02-01

**Authors:** Mariam Bayram, Richard Palluel-Germain, Florent Lebon, Edith Durand, Sylvain Harquel, Marcela Perrone-Bertolotti

**Affiliations:** ^1^Univ. Grenoble Alpes, Univ. Savoie Mont Blanc, CNRS, LPNC, 38000 Grenoble, France; ^2^Laboratoire INSERM U1093 Cognition, Action, et Plasticité Sensorimotrice, Université de Bourgogne, Faculté des Sciences du Sport (UFR STAPS), Dijon, France; ^3^Institut Universitaire de France (IUF), Paris, France; ^4^Département d’Orthophonie, Université du Québec à Trois-Rivières, Trois-Rivières, QC, Canada; ^5^Defitech Chair of Clinical Neuroengineering, Center for Neuroprosthetics (CNP) and Brain Mind Institute (BMI), École Polytechnique Fédérale de Lausanne (EPFL), Geneva, Switzerland

**Keywords:** language, motor imagery, mental simulation, cognitive training, rehabilitation

## Abstract

Studies showed that motor expertise was found to induce improvement in language processing. Grounded and situated approaches attributed this effect to an underlying automatic simulation of the motor experience elicited by action words, similar to motor imagery (MI), and suggest shared representations of action conceptualization. Interestingly, recent results also suggest that the mental simulation of action by MI training induces motor-system modifications and improves motor performance. Consequently, we hypothesize that, since MI training can induce motor-system modifications, it could be used to reinforce the functional connections between motor and language system, and could thus lead to improved language performance. Here, we explore these potential interactions by reviewing recent fundamental and clinical literature in the action-language and MI domains. We suggested that exploiting the link between action language and MI could open new avenues for complementary language improvement programs. We summarize the current literature to evaluate the rationale behind this novel training and to explore the mechanisms underlying MI and its impact on language performance.

## 1. Introduction

In this review, we present theoretical and experimental arguments that show that motor imagery (MI, i.e., a form of explicit motor simulation of action without movement execution) could be used to improve language performance. Firstly, the rationale behind this hypothesis relies on several results showing that the motor system could modulate language processing. For example, motor practice can improve language comprehension in healthy participants (e.g., Beilock et al., 2008; Locatelli et al., 2012), and participants with different diagnoses (e.g., improvement of narrative-understanding in children with dyslexia, Trevisan et al., 2017; and of aphasia batteries scores in post-stroke patients, Harnish et al., 2014). Secondly, a significant body of literature showed the benefits of MI practice on motor functions and motor-system plasticity (Simonsmeier et al., 2020, for a review). Therefore, we hypothesize that modifying the motor system with MI training may lead to language processing improvement akin to those observed following an actual practice. The idea is not novel, as a similar rationale was applied for the endorsement of motor-based protocols for language improvements (e.g., Difrancesco et al., 2012; Chen et al., 2019), and to a combination of action observation, MI training, and other language rehabilitation techniques (Durand et al., 2021). However, it has not been applied to MI alone, and studies never discussed the potential role of MI in these motor-based protocols.

In the present review, we argue that using MI specifically would be beneficial for language processing, based on theoretical, behavioral, neuroimaging, and neurostimulation evidence. To first develop this idea, we focus on the implications of the interaction between motor and language systems and, more specifically, on the effect of sensorimotor training on language performance. Then, we mention MI practice’s benefits on the motor system and how these benefits are partially comparable to those of motor practice. Finally, we discuss that the proposed cognitive mechanisms could be involved in the action/language relationship (i.e., motor simulation) by presenting theoretical, behavioral, and neural evidence. We identify implicit and explicit motor simulation mechanisms during action observation, a motor-based training previously used in novel language rehabilitation protocols, and during MI practice. We argue that MI could improve the possible mechanisms that underlie the relationship between the motor and language systems.

## 2. Relationship between the motor and language system

A growing debate in the field of cognitive neuroscience is concerned with the nature of language: whether it is embodied (i.e., grounded in sensorimotor areas, Barsalou, 1999, 2007; Hauk and Pulvermüller, 2004; Gallese and Lakoff, 2005; Pulvermüller et al., 2005a; Fischer and Zwaan, 2008; Pulvermüller and Fadiga, 2010; Grosprêtre et al., 2016; Conca and Tettamanti, 2018; Pulvermüller, 2018; Conca et al., 2021), or amodal (i.e., words are represented and processed in an autonomous semantic system separate from modality-specific systems; Fodor, 1983; Mahon and Caramazza, 2008; Caramazza et al., 2014). Regardless of the theoretical conception of language, we capitalize on the behavioral illustrations of the link between the motor and language systems.

### 2.1. Motor training and language performance

Some studies showed that physical training leads to improved performance on language tasks (e.g., Beilock et al., 2008; Locatelli et al., 2012; Trevisan et al., 2017; Wang et al., 2022; see Naro et al., 2021 for a review) while physical deprivation can induce a decline in that performance (Bidet-Ildei et al., 2017). These studies suggested that experience-dependent modifications that occur in the motor system could extend to the language system. For instance, Bidet-Ildei et al. (2017) demonstrated the negative impact of sensorimotor deprivation on language performance. They evaluated language processing before and after a 24-h limb immobilization of the arm. They showed that limb immobilization modified the processing of verbs: the control group processed hand-action verbs faster than foot-action verbs, whereas the limb-immobilization group showed no such effect. Additionally, while the control group had reduced response times specifically for hand-action verbs in the post-session, this effect was absent in the hand-immobilization group. Motor activity reduction by limb-immobilization increased language processing times for limb-related verbs specifically.

The beneficial impact of sensorimotor experience on language performance was observed, for example, by Beilock et al. (2008). The authors recruited hockey players, fans, and novices who completed a language comprehension task (i.e., sentence-picture matching task). The stimuli in the task consisted of action sentences describing hockey actions and everyday actions, and participants listened to these same sentences during an fMRI session. While it seems apparent that performing and rehearsing actions will improve their execution in the future, such as in hockey players, authors hypothesized that specialized sports experience also could enhance action-related language understanding. Results revealed an action-match effect for sentences describing everyday actions in all participants and a hockey-action match effect for hockey players and fans only. The results indicated that experience watching and playing hockey facilitated the comprehension of hockey-related action sentences in specific. Neuroimaging data allowed Beilock et al. (2008) to evaluate brain modification induced by such sensorimotor experience (i.e., observation in the case of fans and execution in the case of players). Specifically, they showed that, during language tasks, in experts and fans, the dorsolateral premotor cortex was involved, although the task did not require an intention to perform a real action. The authors claimed that the mediation analysis described a causal chain of events. Sensorimotor experience seems to modify the sensorimotor areas’ degree of involvement during auditory language processing. The results showed that experience-dependent recruitment of motor areas is an integral part in the comprehension of action language (see Wang et al., 2022, for similar, more recent results).

Another example of motor expertise’s effect on language processing comes from the experiment by Locatelli et al. (2012). They recruited naive participants and trained them to perform complex novel manual actions (e.g., origami). They compared their performance on language comprehension tasks before and after the manual training. They observed a significant improvement in processing manual-action-related verbs compared to semantically unrelated action verbs. As sensorimotor expertise seems to improve conceptual processing selectively, it seems that conceptual knowledge accessed during language tasks involves the reactivation of sensorimotor processes.

Another result that corroborated this conclusion was observed by Trevisan et al. (2017), whereby a group of children with dyslexia participated in a study investigating the effect of motor training on language comprehension. According to the authors, the potential comprehension deficits of children with dyslexia could allow more room for post-training improvements. Hence, children with dyslexia listened to action and non-action narratives before (baseline measure) and after engaging in video game-based body training. As a measure of assessment, the children responded to questions about abstract and action verbs embedded in the narratives. Following the video-game-based body training, participants’ demonstrated a boosted comprehension of action-information. It is important to note that the movements described in the narratives did not match those performed during training. As such, results suggested that training effects were not exclusive to effector or movement direction. The results observed by Trevisan et al. (2017) resonate with recent findings that show that MI ability in children with developmental dyslexia correlated with word reading and phonological awareness abilities (van de Walle de Ghelcke et al., 2021, see also section 5 below).

Motor training’s effect on language was examined in clinical contexts as well. For example, Harnish et al. (2014) evaluated language-and motor-fMRI activity in patients with chronic left-hemisphere strokes who received motor therapy without language therapy. Patients’ performance on aphasia batteries and their upper-extremity’s functioning were assessed pre- and post-training. The patients also underwent fMRI during the execution of motor tasks. They showed that the extent of motor and language improvement covaried, as language changes co-occurred with motor changes, and fMRI analysis revealed distinct motor activation patterns in these subjects, marked by a shift in activation toward the right hemisphere. These preliminary results, observed in stroke patients, are a proof-of-concept supporting the direct effect of motor training on language skills, even without language intervention or practice.

Beyond language processing improvements, Vukovic and Shtyrov (2019) showed that sensorimotor training could aid noun encoding and retrieval. In this study, groups of participants received transcranial magnetic stimulation (TMS) 5 min before a word-learning task (action verbs and object nouns). The neuromodulation was inhibitory (i.e., interfered with the learning process), was delivered to the left primary motor area (M1), and compared to an active control and sham control TMS. Kinematic and behavioral measures illustrated the inhibitory effects of TMS. The results suggested a causal role of M1 in the word-learning process, as disruption of M1 directly and negatively affected the learning process compared to the control conditions. Results further revealed a category-specific effect. Indeed, neuromodulation modified the action-word processing only. This study suggested that the motor system could be involved in action-verb encoding and representation. More recently, Mathias et al. (2021) employed another neuromodulation protocol: offline, inhibitory continuous theta burst stimulation (cTBS) combined with repetitive TMS (rTMS). The experiment involved sensorimotor-enriched training for foreign-language learning. The words presented during training were either accompanied by complementary gestures (i.e., sensorimotor-enriched training protocol) or pictures (i.e., sensory-enriched training protocol). The task required participants to translate the newly acquired words while receiving rTMS to M1. Compared to sham stimulation, verum stimulation slowed down the translation process for sensorimotor-enriched foreign words but not sensory-enriched foreign words. The specific influence of rTMS on the translation of sensorimotor foreign words suggested that M1 may be specifically implicated in the representation of these words (see also Tian et al., 2020; Monaco et al., 2021, for similar results, and Adams, 2016, for a review).

Taken together, these studies illustrate the relationship between the motor and language systems. They also suggest that the representations of actions in the motor system are shared with those that subside action, or rather, verb meanings. The latter is corroborated by neuroimaging studies that show the motor system’s somatotopic activation during action-language processing (Aziz-Zadeh et al., 2006; Boulenger et al., 2009; Tomasino et al., 2012; Klepp et al., 2014, 2015; Dreyer and Pulvermüller, 2018; Afonso et al., 2019). The results support the suggestion that motor-based-training can improve language processing. The following sections present how MI can modify the motor system in a specific, somatotopic manner and may thus be used to modify language processing.

## 3. MI training and motor system

The findings summarized in the section above encourage the idea that motor system training may influence language processing. These findings open new avenues in management of language pathologies or atypical developmental conditions, as well as a complementary tool for general language improvement. Furthermore, several results suggested that motor simulation by MI training contributes to enhanced motor skill learning and performance (Schuster et al., 2011; Simonsmeier et al., 2020; Ladda et al., 2021). Based on that evidence, we suggest MI training could also improve language performance.

### 3.1. MI practice and motor system modifications

MI practice describes the repetition of imagined movements in the context of training and motor learning methods (Ladda et al., 2021). MI practice is currently considered as a relevant adjuvant method to physical practice, to further improve motor skills and outcomes in healthy individuals (Guillot and Collet, 2008; Simonsmeier et al., 2020; Ladda et al., 2021) and in clinical populations (Malouin et al., 2013; Monany et al., 2022). In a recent meta-analysis, Simonsmeier et al. (2020) supported the significant benefits of MI practice on motor performance and motivational and affective outcomes (see also O’Shea and Moran, 2019). Specifically, MI practice can improve motor sequencing (Gentili et al., 2010), aiming (Kim et al., 2014), motor timing (Pascual-Leone et al., 1995), strength (Lebon et al., 2010), and motor system flexibility (Guillot et al., 2010; see Ladda et al., 2021, for a review). In terms of training outcomes, MI and physical practice were both shown to induce use-dependent plasticity following equivalent training periods (Ruffino et al., 2019). For instance, a study evaluated MI practice’s ability to improve upper and lower limb strength in healthy adults (Lebon et al., 2010). Results showed that, following training and compared to a control group, participants trained with MI had higher maximal voluntary contraction of lower limb exercises (Lebon et al., 2010). Interestingly, physical practice combined with MI would be more effective than physical practice alone, for the same amount of repetitions, with the intensity of imagery training as a moderating factor, whereby the subjective intensity correlates with objective intensity measures (Simonsmeier et al., 2020). Accordingly, some studies show that athletes who practice using MI have more refined and elaborate mental representations of actions compared to athletes who do not practice using MI (Ingram et al., 2016; Moran and O’Shea, 2020; Simonsmeier et al., 2020, for a review). In an fMRI study of the functional-neuroanatomical networks of MI skills, Guillot et al. (2009) showed that experts were likely to automatize the imagined movements, similarly to when they rehearse them physically. Likely, experts, compared to non-experts, require less cognitive effort (Guillot et al., 2009). These results further consolidate the finding that MI operates at a higher-order level (Mizuguchi and Kanosue, 2017; Moran and O’Shea, 2020). Accordingly, a resting-state fMRI study compared brain changes following MI or physical practice (Kraeutner et al., 2022). Results revealed that MI elicited widespread changes in resting-state activations within a bilateral frontoparietal network. In comparison, physical practice led to focal changes in rs-fMRI limited to a cerebellar-cortical network. The authors suggested that these results indicated that MI practice induces a functional reorganization that may drive motor memory consolidation and learning (Kraeutner et al., 2022). Interestingly, the activation during MI is muscle-specific (Fadiga et al., 1998; Facchini et al., 2002; Stinear and Byblow, 2003), time-locked (Fadiga et al., 1998; Stinear and Byblow, 2003), and dependent on the content of the imagined action (Stinear et al., 2006; Mizuguchi et al., 2013). Moreover, TMS studies (e.g., Ruffino et al., 2019; Yoxon and Welsh, 2020) show that the corticospinal motor system’s activation during MI practice correlated to the magnitude of motor-cortical adaptations following MI training (Ruffino et al., 2019; Yoxon and Welsh, 2020). At the behavioral level, the improvements in the TMS-evoked movement following MI training seem direction-specific and return to baseline 30 min after the training phase, matching results from physical practice protocols (Ruffino et al., 2019).

MI is a promising intervention from a neurorehabilitation perspective (Ruffino et al., 2019). A large number of evidence documents the successful use of MI in the rehabilitation of clinical populations. For instance, MI practice has shown promising results in the rehabilitation phase following surgery for torn anterior cruciate ligament, potentially by increasing muscle activation (Lebon et al., 2012). Studies with chronic stroke patients have shown that MI training for patients suffering from persistent motor weakness can aid the recovery of hand functioning, likely by stimulating the reorganization of brain activity (Ietswaart et al., 2006, 2011; Mateo et al., 2018; Yoxon and Welsh, 2020).

## 4. Motor simulation: A possible mechanism underlying language modifications following motor training

Neuroimaging and behavioral methods tend to show that MI may modify the motor system’s activity. Therefore, MI appears to be a solid candidate for a complementary tool for language-processing improvements. To support this idea, the mechanisms that underlie its effects on the motor system deserve attention. For instance, for MI to induce similar modifications to language as physical practice does, it should rely on brain networks and mechanisms shared with those that underlie physical practice. Following, we present some theories of these mechanisms.

### 4.1. Motor simulation and motor imagery

Motor simulation is potentially the mechanism underlying the functional modifications brought to the language system by motor training. Motor simulation entails the mental rehearsal of a motor task that could be initiated explicitly or triggered implicitly (Jeannerod and Frak, 1999). Implicit motor simulation is thought to occur when subjects covertly simulate action during the performance of another task and in the absence of an instruction to do so (Jeannerod and Frak, 1999).

According to motor simulation theory (MST; Jeannerod, 1994, 2001, 2006), rehearsing an action is possible overtly and covertly through action observation and MI. Within this theory, simulation refers to the offline rehearsal occurring in neural networks, and the activation of the motor systems is, therefore, prerequisite for simulation (Jeannerod, 2001, 2006). These two forms of cognitive simulation can activate motor regions of the brain similarly to actual physical execution (Hardwick et al., 2018). An ensuing proposal is that MI shares certain mental representations and mechanisms with action execution, which would explain why MI activates neural pathways shared with action execution. Furthermore, according to MST, MI works by rehearsing neural motor networks through a hypothesized simulation process. Essentially, the theory argues that imagined movements entail the internal simulation of actual movement. In the covert stage, actions are considered to be mentally simulated. The simulation process entails the objective of the action and its consequences (i.e., anticipation). In this manner, the intention to act underlies both represented (i.e., imagined) and actual movements, and that intention would be converted to actual execution in the case of overt actions only. In line with this tenet of MST is the documented functional equivalence between simulation and action performance. The latter implies that imagined and executed actions would be assigned to common motoric representations (Jeannerod, 1994). Following this logic, MI ought to involve neural mechanisms similar to those that underlie actual actions (Jeannerod, 2001) and possibly the meaning of verbs referring to these actions. The latter proposal is extended within the functional equivalence hypothesis (Moran et al., 2012), and corroborated by behavioral and neuropsychological evidence (Moran and O’Shea, 2020) showing, for instance, a significant resemblance between the duration of imagined and actual movements (Guillot and Collet, 2005, for a review; Papaxanthis et al., 2002). Additionally, the neuroplasticity induced by MI practice (see the previous section) is indicative of motor commands issued during MI, which have the capacity to influence spinal cord activity (Debarnot et al., 2014 and Grosprêtre et al., 2019, for a review). In further support of the stipulation that MI is the emulation of the process of execution, we recall the documentation of enhanced and muscle-specific corticospinal excitability during MI (Fadiga et al., 1998; Mizuguchi and Kanosue, 2017).

Internal forward models are theorized to support the ability to simulate actions mentally. Specifically, it is held that the brain uses forward modeling to predict the consequences of action (Miall and Wolpert, 1996; Wolpert, 1997; Wolpert and Flanagan, 2001; Imamizu and Kawato, 2009). In this framework, when motor commands are sent to the motor system to achieve a particular intended end-state, an efference copy is issued in parallel. This efference copy (see Sperry, 1950) is then used to compute a prediction (i.e., a corollary discharge) of the sensory outcome of the motor plan. It is held that motor prediction *via* forward models ensures the production of fast and accurate movements and the prediction and cancelation of the sensory consequences of overt and covert mental actions (Wolpert et al., 1995; Wolpert, 1997; Wolpert and Flanagan, 2001; Gueugneau et al., 2015). As to MI, forward internal models may underlie the temporal features of mental movements, which are equivalent to actual actions (Papaxanthis et al., 2012). Since MI entails no action execution and only muscle preparation, it was proposed that the simulated motor commands’ efference copy remains available to the forward models. In turn, these models provide temporal information that is highly analogous to the information provided by actual actions. The latter is supported by studies that show the duration equivalence between imagined and executed movements.

Interestingly, the study by Beilock et al. (2008) (see section “2.1. Motor training and language performance”) demonstrated that both action execution (i.e., hockey players) and action observation (i.e., fans) led to experience-dependent functional modifications in behavioral performance and neural substrates. Principles of action-perception coupling and internal forward models potentially could explain this result. Correspondingly, neuroimaging studies showed that action execution and action observation induce an overlapping activation of several brain regions also shown to have been recruited during MI (Buccino et al., 2001; Hardwick et al., 2018; Courson and Tremblay, 2020). These results were in line with the hypothesis of a simulation process, the mental rehearsal of actions that occurs in the same brain regions that underlie effective actions.

These stipulations that rely on simulation processes claim that simulated and exerted actions share partially overlapping representations. Therefore, they support the hypothesis that MI can be employed to improve language processing, with the expectation of effects similar to those observed after physical practice (see section “2.1. Motor training and language performance”). Consequently, if actions and verb meanings that refer to those actions share common representations (see section “2.1. Motor training and language performance”), MI would again be considered a candidate for language processing improvements. Following, we outline the possible role of motor simulation in language processing.

### 4.2. Motor simulation and language processing

When it comes to language processing, the specific role of motor simulation mechanisms is not fully understood. It is suggested that extensive motor experience may trigger implicit motor simulation, which allows the linguistic association of an action scene with motor execution plans (Barsalou et al., 2008; O’Shea and Moran, 2017). However, the definition of when and how motor simulation may occur is unclear.

A growing body of literature suggests that motor training may support action understanding. This pattern is predicted by principles of action-perception coupling (Billard et al., 2020), whereby action perception and action execution would be coupled intrinsically by an association between movements and their predicted effects. In this manner, perception of an action’s effect can elicit a representation (or simulation) of the action required to reach that effect. Movement perception can thus trigger perceptual processes (Wolpert and Kawato, 1998; Novembre and Keller, 2014). Numerous findings have also shown a co-dependency between action and language, whereby language seems to boost action-perception coupling (Borghi and Cangelosi, 2014; Billard et al., 2020, for experimental evidence, see studies on the action-sentence compatibility effect, ACE, Aravena et al., 2010; Ibáñez et al., 2013).

In a study of motor expertise’s effect on language comprehension, Tomasino et al. (2012) examined if motor simulation occurs only when action language refers to possible actions (i.e., that can be physically experienced), or also when the action language is semantically correct but refers to impossible actions. The authors explored how expert volleyball players, fans, and novices decide whether sentences they read referred to a possible action. The experimental task contained volleyball-specific action sentences and semantically correct sentences that described impossible actions. Additionally, the sentences contextually varied, and were phrased negatively (e.g., “Don’t shank”) or positively (e.g., “Assist”). At a behavioral level, previous studies showed that negative imperatives, compared to positive imperatives, result in relatively longer response times. At a neural level, it is likely because negative contexts reduce implicit simulation (Tomasino et al., 2014a,b), or require inhibition of simulation mechanisms (Foroni and Semin, 2013; Tomasino et al., 2014b). Results revealed that, athletes and fans took a longer time to process negative sentences compared to positive sentences, and this difference was greater for possible actions compared to impossible actions. Overall, experts were faster and more accurate than fans, who were also more accurate than novices. According to the authors, an implicit motor simulation may affect action-verb processing depending on expertise, action feasibility, and negative context. Results from Tomasino et al.’s (2012, 2014b) study highlighted how motor simulation mechanisms could play a role in the link between action and language. More specifically, these studies suggested that action word processing could induce the mental simulation of such action that involves not only language networks but also neural networks effectively involved during the effective action execution. However, it could be that participants in that study were engaging in MI during the task. Indeed, MI is itself a form of motor simulation, albeit voluntary. Di Gruttola (2018) interviewed their participants about the strategies they adopted during motor tasks. Half the participants claimed to have spontaneously engaged in concurrent MI. In another study by Meers et al. (2020), participants were instructed specifically not to engage in concurrent MI, which led to a change in brain patterns (see Vogt et al., 2013, for a review). Likewise, the primary visual cortex is active during MI, and was showed to be specifically more active during MI of complex body movements [e.g., impossible movements, similar to those prompted by Tomasino et al.’s (2012) task] compared to simpler body movements (Mizuguchi et al., 2016), as it is during action observation compared to MI. MI of experts is more evolved than it is for fans or novices, as they perform it more often than amateurs and can associate it with physical sensations (Cumming and Hall, 2002; Guillot and Collet, 2008; Mizuguchi and Kanosue, 2017). In the same vein, other findings showed that, during MI, experts exhibited more activity in their motor cortex, compared to non-experts (Mizuguchi and Kanosue, 2017). Additionally, fans do not physically rehearse actions but only engage in action observation, and they may not associate the observed actions with their own physical sensations. Indeed, MI quality improves by appropriate sensorimotor signals, which experts experience more frequently than fans (Mizuguchi et al., 2009, 2012). It is possible that, in the study by Tomasino et al. (2012), the experts’ advantage over fans (in speed and accuracy) could be explained by their physical expertise, as well as by their superior MI abilities. These superior abilities could have also led to the longer processing times for negative sentences compared to positive sentences for possible actions (i.e., superior MI abilities may have caused an interference during the processing of negative sentences referring to possible actions).

A notable amount of research revealed that neuronal networks engaged during imagined and executed movements overlap (Ehrsson et al., 2003; Guillot et al., 2009; Hardwick et al., 2018). Some of these could also have been activated during the passive reading of action verbs. Yang and Shu (2014) examined the fMRI brain activity of participants during a reading task (hand action verbs and two types of tool-use verbs), while they imagined themselves executing these actions. Results revealed that all verb types activated similar patterns of brain networks in the MI and passive reading conditions (e.g., in hand motor areas). However, compared to passive reading, MI of the same action verbs produced more activity in the superior frontal gyrus, supplementary motor area, and cingulate cortex (see also Tomasino et al., 2008). The activation exhibited in these regions during the MI task compared to the passive reading task could indicate that the motor simulation mechanisms that occur during MI involve more cognitive regulation, motor planning, and coordination between motor and sensorimotor systems than those in passive reading. More specifically, MI activates a motor-related network, such as M1, the pre-frontal cortex (premotor cortex and supplementary motor area), the parietal lobe, the cerebellum (implicated in cognitive aspects of motor control and internal bodily representation), and the putamen (a structure involved in automatic and rapid movements, Pelgrims et al., 2011; Hardwick et al., 2018). Some of these areas are relevant in the processing of action language. For instance, M1 and the prefrontal cortices have been found to be involved during action-language processing (Hauk et al., 2004; Yang and Shu, 2012; de Vega et al., 2014). Durand et al. (2021) suggested that the motor and the premotor cortices, the visual network, and the language system, together support the functional relationship between language and action. Furthermore, MI was previously showed to activate the inferior frontal gyrus (Vogt et al., 2013), a brain area that has been found to be involved in language comprehension (Ishkhanyan et al., 2020). Incidentally, the ability to perform MI was also found to be associated with activity in the frontoparietal motor areas (Guillot et al., 2009).

It remains unclear whether the sensorimotor mechanisms that occur during language processing (i.e. motor simulation) are involved in the early or late stages of action-word processing (Hauk and Pulvermüller, 2004; Pulvermüller et al., 2005b; Boulenger et al., 2006; Simmons et al., 2008; Papeo et al., 2009, see next section for more details). An early implication of the motor system, *via* motor simulation, is in line with the statement that the motor system plays a crucial role in facilitating lexical-semantic access (Pulvermüller et al., 2005a; Boulenger et al., 2006). Conversely, other authors proposed that the involvement of the motor system is an epiphenomenal event that results from post-conceptual access (Mahon and Caramazza, 2008; Papeo et al., 2009, for a discussion). Evidence for the timeframe of the motor system’s implication in language processing comes from TMS studies. TMS studies typically examine the Motor Evoked Potentials (MEPs) from targeted muscles to directly measure the excitability at pulse delivery time during a given behavioral task. The MEP amplitude following a TMS pulse is proportional to the activity level in the stimulated brain area at a specific timeframe (Papeo et al., 2009). Studies that employ similar methodology found that M1 is active in late phases of semantic processing, around 400–500 ms after verb presentation, rather than during the early stages of semantic processing (170–350 ms post-stimulus; Papeo et al., 2009). However, results from other TMS studies go in line with the claim that the motor system’s activation during action-concept processing is rapid and automatic: action-verb presentation has been found to modulate MEPs during semantic tasks in early processing timeframes (200–300 ms post-stimulus; Buccino et al., 2005; van Elk et al., 2010; Innocenti et al., 2014; De Marco et al., 2018; Reilly et al., 2019). Studies employing rTMS reported that the processing of action verbs referring to a given limb is disrupted by rTMS to the limb’s cortical representation in M1, while the processing of other action words was spared (Kuipers et al., 2013; Repetto et al., 2013; Vukovic et al., 2017). The latter implies the presence of a shared neural representation of the limb and its semantic representation. The disruption in processing was evident not only in modulated RTs but in modulated event-related potential (ERPs) measured using EEG coupled with TMS (Kuipers et al., 2013; Vukovic et al., 2017). Likewise, the neuromodulation of M1 using transcranial direct current stimulation (tDCS) can selectively improve performance in a task that requires the comprehension and memory of action sentences, indicating that M1 is implicated causally in the processing of linguistic meanings (Vitale et al., 2021). Combined with the results of behavioral motor training, these motor-stimulation studies highlight a bidirectional relationship that connects the language and motor systems and underlines a causal role of motor activity in language processing. They also suggest that improving motor simulation networks may improve language processing.

In sum, motor simulation processes could explain the pattern of results in most of the studies summarized above. The application of neurostimulation in a motor region during implicit simulation delivered during action language processing causes a modification of language performance. We established the evidence supporting the relationship between language and motor system, and MI and the motor system. We also mentioned how MI may have played a role in the findings above summarized. Following, we outline more explicit evidence of the direct role of MI in language processing that may suggest the use of MI as a tool for improving language in various cases.

## 5. MI and language processing

We mention again the limb-immobilization study (Bidet-Ildei et al., 2017, see section “2. Relationship between the motor and language system”), in further support of our rationale. The study highlighted a relevant result: regardless of the hand-immobilization condition, participants with the highest MI (i.e., overt motor simulation) capacities demonstrated shorter response times for all hand- and foot-action verbs. The correspondence between MI capacity and processing time of action verbs highlights an underlying mechanism that could explain the overall pattern of results and shows the involvement of an overt mode of motor simulation (i.e., MI) in language processing. Further experimental evidence highlights the direct role of MI in language processing.

### 5.1. Experimental evidence of the role of MI in language processing

In a recent and related proposal, Cayol and Nazir (2020) suggested that language processing involves modality-specific brain areas for predictive purposes. The processing involves emulators: a mechanism that learns the causal relationship between action and its sensory consequences, evolved for motor control, to assess the situation illustrated by the verbal stimuli. The recruitment of emulators engages associative-memory networks supporting semantic processing. To test this hypothesis, Cayol et al. (2020) aimed to establish a link between MI task and performance in a word definition task in adolescents. Results revealed that MI aptitude predicted language processing skills. The latter finding resonates well with the representative nature of MI (Moran and O’Shea, 2020). MI aptitude’s correlation with language processing skills could be illustrative of a better representation of the action concepts in the brain.

If we follow the rationale of theories on motor simulation mechanisms, we would expect the correlations between MI and language processing, especially considering that representations of actions and their corresponding verbs’ are shared. An explicative hypothesis is a bilateral interaction between language and motor systems, as reflected by results showing that disturbances to the language system induce modifications in the motor system, and vice versa.

Interestingly, disturbances to MI have been documented in dyslexia. Dyslexia is a severe and frequent disorder of reading acquisition. It is accompanied often by cognitive and motor difficulties, namely in automatization of sensorimotor control. According to the sensorimotor perspective on dyslexia (Stein, 2001), phonological deficits, which are prevalent in the disorder, may be a manifestation of a larger, multimodal syndrome. van de Walle de Ghelcke et al. (2021) were interested in the internal action representation in dyslexia. The internal action representation is theorized to be a form of prediction—a function of forward internal models—that allows sensorimotor control. Indeed, several results suggested impairment in balance control, motor coordination, and motor learning in dyslexia (Stoodley and Stein, 2013). This pattern of evidence alludes to a link between motor and reading mechanisms, possibly including the ability to mentally represent actions since it constitutes a crucial feature of motor behavior (van de Walle de Ghelcke et al., 2021). These findings inspired the authors to explore the presence of deficits in action representation in developmental dyslexia. Their study included a group of adolescents with developmental dyslexia without associated diagnoses and age-matched typical readers. These participants completed, mentally and physically, a visually guided pointing task. The task involved spatiotemporal constraints and required a speed-accuracy trade-off. The estimation of mental-action representation was done in relation to Fitts’ law, which predicts that the time taken to perform a movement would increase linearly with task difficulty (Fitts, 1954). In addition, it was estimated based on the isochrony between actual movement time and mental movement time. Authors hypothesized that task difficulty would modulate the typical readers’ imagined and actual movement times (i.e., following Fitts’ law). However, that modulation would not occur for participants with dyslexia due to deficits in action representation. Results revealed that the group with dyslexia performed mental and actual movements slower than the control group. Results also showed a deficit in mental actions in the group of participants with dyslexia as estimated by Fitts’ law in the mental action condition. Overall, these results supported the presence of deficits in action representation in dyslexia. In section “2.1. Motor training and language performance,” we mentioned the study by Trevisan et al. (2017), that showed that sensorimotor training could improve language processing in children with dyslexia. Taken together, the presence of MI deficits in dyslexia, and the ability of sensorimotor training to improve language processing in dyslexia, point to a shared mechanism and representation between motor and language systems. MI practice, therefore, comes out as a strong practice for language-processing improvements in the case of dyslexia.

While MI seems to be related to language-related deficits such as dyslexia, it also appears that MI practice can improve the ability to understand spoken, action-related instructions. In a recent study, children listened to action-object commands and encoded them by MI or verbal rehearsal. When asked to recall the instructions, children who practiced them using MI showed superior recall abilities compared to children who verbally rehearsed the instructions. Results suggested that MI may be a strategy for learning spoken instructions (Yang et al., 2020) and for an overall improvement in language processing.

We hypothesize that MI training could be employed to strengthen the internal and cortical representations of action concepts (Hauk et al., 2008; Tomasino et al., 2008), since it can lead to changes in higher-order representations of actions (Mizuguchi and Kanosue, 2017; Savaki and Raos, 2019; Moran and O’Shea, 2020) as well as to reinforce language and motor brain network connectivity. This suggests that MI is a candidate for a scope of language processing improvements, including learning new words and foreign words (see section “2.1. Motor training and language performance” for information on sensorimotor training for foreign-word acquisition).

We recently proposed an MI-training protocol for healthy adults and evaluated language comprehension before and after training (Bonnet et al., 2022). Participants performed a semantic categorization task where they categorized verbs–as quickly and accurately as possible–as concrete or abstract. The verbs in that task were abstract or action verbs (referring to the upper- and lower-limb actions). The MI-training protocol did include some of the upper-limb action verbs. Compared to a control, static-visual imagery training group, participants in the MI-training group showed faster responses for all verb categories (i.e., trained and untrained action and abstract verbs). To our knowledge, these results showed for the first time that MI could improve the processing time of verbs in a group of healthy adults (Bonnet et al., 2022).

Overall, the body results suggested that MI training can improve language comprehension of action and abstract language. Tomasino et al. (2007) investigated whether MI occurs automatically or when explicitly solicited during language processing. In an fMRI study, participants read short sentences that were either motor-related or non-motor-related. In a second task, participants explicitly imagined the situation or performed a letter detection task (preventing them from simulating). Results from fMRI showed that, in the imagery condition, M1 activity is enhanced when motor-related sentences at the presentation of motor-related sentences. Here, the somatotopic activation in motor cortices may have been due to implicit simulation strategies adopted by participants. Likewise, a study by Tomasino et al. (2008) highlights both the neural and behavioral implications of MI in semantic processing. Participants silently read verbs in three conditions: one where they simply indicated if they finished reading, another where participants judged whether the corresponding movement involved hand rotation (i.e., MI condition), and the last condition in which they made a judgment on word frequency (Tomasino et al., 2008). It appeared that TMS to M1 differentially modulated participants’ performance. The TMS showed a facilitation in only the MI condition, suggesting that M1 is crucially involved in processing action verbs only when participants simulated the corresponding movement.

In sum, even if it remains unclear whether patterns associated with motor simulation and MI are similar or essentially distinct, the proposal of MI as a possible language rehabilitation technique remains compelling. Indeed, if MI’s effects on the motor system are comparable to those of actual action execution due to the plastic effects that it induces in relevant brain areas by virtue of its long-lasting effects on M1’s excitability and activity (Ruffino et al., 2021). Moreover, evidence points to its involvement in language processing (Tomasino et al., 2008; Cayol and Nazir, 2020). Additionally, MI does share some neural representations with implicit motor simulation, a process that can modulate language processing (e.g., Tomasino et al., 2012, 2014a; Yang and Shu, 2014). However, not all results support the latter (Willems et al., 2010).

## 6. Language processing improvement by motor and MI training: Existing protocols

The results, summarized above, encourage motor training to improve language processing. The motor and language relation has inspired several rehabilitation protocols based on the motor system. Indeed, examples of such motor-based rehabilitation protocols for language are available in the literature and have shown promising results with clinical populations (e.g., stroke patients, MacGregor et al., 2012; Chen et al., 2019; or post-stroke aphasia patients, Difrancesco et al., 2012; or patients of neurological disorders with verb anomia, Durand et al., 2018, 2021; or Alzheimer’s patients with limb apraxia; Cotelli et al., 2014). Following, we summarize the existing protocols that rely on action observation.

### 6.1. Action observation rehabilitation protocols

“Intensive Language Action Therapy (ILAT)” (Difrancesco et al., 2012) is an aphasia rehabilitation program based on the connection between sensorimotor and language systems. ILAT was developed based on principles of neuroscience and corroborated by scientific evidence (Pulvermüller and Berthier, 2008; Berthier and Pulvermüller, 2011). ILAT is a set of techniques recommended for speech and language therapy, emphasizing intensive and frequent practice (Difrancesco et al., 2012) and the use of action-embedded language pertinent to daily life, embedded in personalized treatment plans. ILAT seemed to be effective in treating chronic aphasia. Patients who received ILAT showed improvements in language abilities such as auditory language comprehension, speech production, and communicative performance. ILAT led to an increased activation in perilesional areas (Difrancesco et al., 2012; MacGregor et al., 2012; Dreyer et al., 2021). The results provide further evidence of the neuroplasticity elicited by motor-based language rehabilitation. While there may be cases where motor execution would be excessively effortful or impossible for patients, alternative motor-based options exist; MI and action observation require almost no effort and share neural mechanisms with effective action execution (Guillot et al., 2009; Collet et al., 2013; Hétu et al., 2013; Hardwick et al., 2018).

Chen et al. (2019) developed a training protocol based on hand-action observation (e.g., repeating “pen” after watching a video of someone writing and then saying “pen”) for aphasia patients and compared its effects to outcomes of conventional speech therapy and a dynamic-object observation training (e.g., watching a dynamic video of a pen and repeated “pen”). fMRI techniques assessed patterns of brain activity during hand action and dynamic-object observation. Results indicated that, compared to baseline assessments, patients in the hand-action observation group improved more than patients in the dynamic-object observation group on the aphasia quotient and its affiliated naming subtests. These patients also demonstrated a higher score on the aphasia battery. The improvement in the hand-action observation condition was comparable to that observed in a conventional speech therapy group. The hand-action observation training correlated with a higher activation of the inferior frontal gyrus (predominantly involved in speech production), the superior temporal gyrus (predominantly involved in speech comprehension), and the supramarginal gyrus (predominantly involved in identifying postures and gestures). Neuroimaging results suggest that the therapy can induce neural plasticity (Chen et al., 2019) and provide evidence that motor-based therapy may be an innovative intervention in post-stroke language rehabilitation (see for similar conclusions Durand et al., 2018, 2021).

The protocols used in the above studies mostly rely on action observation, a process that shares neural substrates with action execution and MI (Hardwick et al., 2018). Namely, motor simulation mechanisms are thought to operate beneath both MI and action observation. However, only one rehabilitation program included MI in its protocol for verb-anomia rehabilitation (Durand et al., 2018, 2021) in combination with action observation. Action observation is a covert form of motor simulation (Jeannerod, 2001, 2004; O’Shea and Moran, 2017). Like action observation, MI partially activates the networks that underlie actual execution (Jeannerod, 2004; O’Shea and Moran, 2017; Raos and Savaki, 2017; Hardwick et al., 2018). However, MI further recruits some brain areas due to its representational nature (O’Shea and Moran, 2017) and because, as a top-down, conscious process, it involves the correct attribution of agency to imagery (Savaki and Raos, 2019; Chye et al., 2022).

### 6.2. MI as compared to action observation

How would MI differ from action observation? Action observation has shown promising results in the rehabilitation of language in patients (Difrancesco et al., 2012; MacGregor et al., 2012; Chen et al., 2019), and so has action observation combined with MI (Durand et al., 2018, 2021). Action observation is, by nature, a bottom-up process that involves the structured observation of human movement (Neuman and Gray, 2013), while MI is a top-down process that requires the internal generation of the visual and/or kinesthetic elements of movement (MacIntyre et al., 2013). The literature has reported positive behavioral outcomes following action observation and MI practice for sports (Guillot and Collet, 2008), and for neurorehabilitation purposes (Buccino, 2014; Simonsmeier et al., 2020). A recent large-scale meta-analysis of fMRI data showed that MI, action observation, and movement execution give rise to a shared network that includes premotor, rostral parietal, and somatosensory areas of the brain (Hardwick et al., 2018). More importantly, the meta-analysis also revealed that motor execution shared more of its underlying areas with those that underlie MI, compared to action observation (Hardwick et al., 2018).

A recent study (Meers et al., 2020) examined the motor simulation processes underlying concurrent action observation (AO) and MI by measuring motor-evoked potentials induced by TMS delivered to the right-hand M1 of neurotypical participants. In a congruent AO and MI condition, participants observed videos of a model’s hand performing rhythmical finger movements while they imagined executing those same movements. Compared to baseline measures, this condition showed strong facilitatory effects only in the effector involved in the task. In comparison, in the incongruent AO and MI condition, participants were to imagine a movement incongruent with the one presented in the video. This condition produced equally strong facilitator effects in the effector engaged in MI only, with no corticospinal facilitation for the effector involved in the action in the video. There was also a trend toward lower motor evoked potentials for the AO component in that condition. The results suggest that AO and MI are not both simulated at the level of M1. It was yet another replication of a finding that engaging in AO without MI does not produce reliable effects and that MI alone can account for the activations during AO combined with MI. The authors attempted to reconcile these results with the literature by citing Di Gruttola (2018), who interviewed their participants after a session of pure AO. About half of their participants claimed to have spontaneously engaged in concurrent MI. Whereas, in Meers et al.’s study (2020), the participants received specific instructions to disengage from MI during the AO-only portion of the protocol, which led to the disappearance of AO effects (for a review, see Vogt et al., 2013). A recent meta-analysis of 34 studies (Chye et al., 2022) that employed TMS during combined AO and MI (usually, the kinesthetic modality of MI). Typically, these studies explore the changes in MEP amplitude across various movements. They consistently showed an increased corticospinal excitability during combined AO and MI compared to the baseline (e.g., Vogt et al., 2013; Bruton et al., 2020). Studies with a similar protocol that aim to contrast combined AO and MI against AO or MI alone have reported either increased (Mouthon et al., 2016; Wright et al., 2016) or similar (Castro et al., 2021) corticospinal activity compared to combined AO and MI. The meta-analysis (Chye et al., 2022) revealed that, compared to AO and MI, AO had a medium positive effect size on MEP amplitudes. As for MI compared to AO and MI, the combination of AO and MI had a medium positive effect on MEP amplitudes compared to MI conditions. Concerning movement outcomes, the combination of AO and MI had no significant influence on movement outcomes compared to AO alone and MI alone. Indeed, these effect sizes are based on studies with varying methods and protocols, and the conclusion should be conservative. The verbs trained in the above rehabilitation protocols are simple, daily action verbs. When considering that MI may occur during action observation, we go back to the studies mentioned in section “2.1. Motor training and language performance” and section “6.2. MI as compared to action observation,” and reconsider the role that MI mechanisms may have played in the language improvements, which is largely attributed to physical practice or action observation mechanisms (see section “5.1. Experimental evidence of the role of MI in language processing”). Yet, it is worth noting that a large body of literature recommends using a combination of AO and MI, for better outcomes and further brain activity (e.g., Vogt et al., 2013; Aoyama et al., 2020). Some studies also found that AO alone is a solid candidate for rehabilitation of motor impairments (e.g., Buccino, 2014), and better for acquiring novel motor tasks (e.g., Bazzini et al., 2022).

## 7. Limitations and conclusion

There are further considerations concerning the design of the MI training protocol for rehabilitative purposes. For instance, studies which investigate training strategies and biomarkers for optimal MI practice are rare (Ladda et al., 2021). Moreover, MI can be affected by motor disorders and brain injuries (Malouin et al., 2008; Liepert et al., 2012; Poliakoff, 2013; Di Rienzo et al., 2014; La Touche et al., 2019), but remains trainable and involves more cognitive strategies (Daprati et al., 2010), and is used frequently as a successful tool for motor rehabilitation (Lebon et al., 2012). Moreover, MI ability varies interindividually, and it was found to correlate with brain activity measured during MI (Lebon et al., 2012, 2018; Zabicki et al., 2017). Nevertheless, evidence suggests that MI ability can be improved with MI practice (Ruffino et al., 2017), meaning that brain activity associated with MI can also increase while it induces plasticity and increasing cortical excitability (Ruffino et al., 2017). Assessing MI as part of a rehabilitation protocol can also be challenging, seeing that MI is composed of various modalities, such as kinesthetic, visual, and temporal (Collet et al., 2011; Williams et al., 2015).

Further considerations recall the principles of rehabilitation that should be implemented within an MI-based protocol. For instance, practicing MI because it allows the exploitation of residual neurological functions (Pulvermüller and Berthier, 2008; Berthier and Pulvermüller, 2011) and can activate the motor system similarly to action execution (Hétu et al., 2013; Hardwick et al., 2018). MI practice should not lead to a neglect of other residual functioning, such as physical execution or neurorehabilitation. Indeed, it is the combination of action execution with mental training leads to a larger improvement in performance (Simonsmeier et al., 2020). However, it can be supposed that MI can replace physical execution when it is very limited or effortful (for instance, in acute phases following stroke).

Finally, for MI practice to further encourage improvement in language, it should occur in an adequate context, such as to boost correlational learning with relevant neuronal activation occurring together (e.g., Pulvermüller and Berthier, 2008). The latter implies that the practice should frequently occur to ensure amassed training and repetition-induced neurogenesis (Kleim and Jones, 2008; Pulvermüller and Berthier, 2008).

## Author contributions

MB, RP-G, FL, ED, SH, and MP-B: conceptualized and wrote the original manuscript and revised and edited the manuscript. MP-B and RP-G supervised the work. All authors contributed to the article and approved the submitted version.
